# Risk factors associated with overweight and obesity among urban school children and adolescents in Bangladesh: a case–control study

**DOI:** 10.1186/1471-2431-13-72

**Published:** 2013-05-08

**Authors:** Mejbah Uddin Bhuiyan, Shahaduz Zaman, Tahmeed Ahmed

**Affiliations:** 1 James P Grant School of Public Health, Dhaka, Bangladesh; 2 Centre for Communicable Diseases, icddr,b, 68, Shaheed Tajuddin Ahmed Sarani, Mohakhali, Dhaka, 1212, Bangladesh; 3 Institute of Health and Society, Newcastle University, Baddiley-Clark Building, Richardson Road, Newcastle upon Tyne, NE2 4AX, UK; 4 Centre for Nutrition and Food Security, icddr,b, 68, Shaheed Tajuddin Ahmed Sarani, Mohakhali, Dhaka, 1212, Bangladesh

**Keywords:** Risk factors, School children, Overweight, Obesity, Physical activity, Sedentary activity

## Abstract

**Background:**

Childhood obesity has become an emerging urban health problem in urban cities in Bangladesh, particularly in affluent families. Risk factors for obesity in this context have not been explored yet. The objective of this study was to identify the risk factors associated with overweight and obesity among school children and adolescents in Dhaka, Bangladesh.

**Methods:**

From October through November 2007, we conducted a case–control study among children aged 10–15 years in seven schools in Dhaka. We assessed body mass index (weight in kg/height in sq. meter) to identify the cases (overweight/obese) and controls (healthy/normal weight) following the Centers for Disease Control and Prevention age and sex specific growth chart. We used a structured questionnaire to collect demographic information and respondent’s exposure to several risk factors such as daily physical activity at home and in school, hours spent on computer games and television watching, maternal education level and parents’ weight and height.

**Results:**

We enrolled 198 children: 99 cases, 99 controls. Multiple logistic regression analysis revealed that having at least one overweight parent (OR = 2.8, p = 0.001) and engaging in sedentary activities for >4 hours a day (OR = 2.0, p = 0.02) were independent risk factors for childhood overweight and/or obesity while exercising ≥ 30 minutes a day at home was a protective factor (OR = 0.4, p = 0.02). There were no significant associations between childhood overweight and sex, maternal education or physical activity at school.

**Conclusion:**

Having overweight parents along with limited exercise and high levels of sedentary activities lead to obesity among school children in urban cities in Bangladesh. Public health programs are needed to increase awareness on risk factors for overweight and obesity among children and adolescents in order to reduce the future burden of obesity-associated chronic diseases.

## Background

Childhood obesity is one of the major public health problems globally. During 2005, the prevalence of childhood overweight and obesity in high-income countries was 10.6% whereas in low-income countries, the prevalence was 5.2% [1-3]. Data from high- and low-income countries suggest that lack of physical activity, spending time on sedentary activities such as watching television and playing video games, low parental education, and family history of obesity are risk factors for childhood overweight and obesity [4-9]. Childhood obesity is a risk factor for several chronic diseases such as hypertension, type 2 diabetes, respiratory disease, and hepatic abnormalities and coronary heart diseases during adulthood [10,11]. Additionally, overweight and obesity affect self-esteem of children and impair social development [12,13].

In Bangladesh, nearly 40% of children < 5 years are suffering from malnutrition [14]. However, in recent years, multiple factors such as rapid urbanization, continually decreasing number of playgrounds, increasing purchasing power, and easy access to new technological devices such as hand-held computer toys probably have lead to less physical activity and more sedentary activity, and thereby have attributed to an emerging overweight and obesity problem among young children in urban settings, especially among affluent families in Dhaka [15]. The country is now experiencing the double burden of malnutrition, with the existence of both undernutrition and overnutrition, in urban cities. Childhood overweight and obesity is a particular public health concern for Bangladesh because children who are overweight or obese have higher risk of becoming overweight or obese adults [16,17] and overweight adults are at increased risk for mortality and morbidity with obesity-associated chronic diseases, which are already a burden to the struggling health system in Bangladesh [18,19].

Several studies suggest that schools are a potential setting to target both children and adolescent population for obesity prevention [20,21]. Identifying risk factors associated with overweight and obesity in school children would help to develop appropriate interventions to reduce the future burden of overweight and obesity among young population in Bangladesh. The objective of this study was to describe the risk factors associated with overweight and obesity among school children in Dhaka, Bangladesh.

## Methods

### Study sites

We conducted this study in seven schools located in Dhanmondi, Mohammadpur and Siddeswari area in Dhaka, Bangladesh during October through November 2007. The highest numbers of schools in Dhaka are situated in these places. We selected these schools based on three criteria: large number of students, having reputation for better education facility, and availability of play-ground within school premises. Each of seven schools was private and English was the medium of teaching in three of seven schools.

### Study design and study participants

The study was designed as case–control study with cases being overweight children^a^ and controls being healthy/normal weight children. The study participants were students of age 10-15 years corresponding to class 5 to class 10 in the selected schools.

### Sampling size estimation

We used *EPI Info* version 3.3 to calculate sample size for this study. Considering 80% power, 95% confidence level and case–control ratio 1:1, we calculated that 240 participants were required to detect at least 2 odds ratio differences between cases and controls.

### Case–control identification

We adopted purposeful sampling method to select the cases and controls. The teachers at each school made initial selection of 12–15 cases by visually assessing whether the students were overweight and also selected equal number of controls who visually seemed normal weight. Then, research assistants used standard procedures of anthropometry to measure standing height and weight (including shoes) of each primary selected participant and calculated body mass index (BMI) as weight in kg/height in meter^2^. Investigators used age- and sex-specific growth chart by the US Centers for Disease Control and Prevention [22] to categorize initially selected participant as cases or controls. Any participant falling into the underweight category according to growth chart was excluded from the study. We selected equal numbers of cases and controls from each school.

### Data collection

We used a structured questionnaire to collect demographic information and information on exposures of interest: regular habit of physical activities, duration of sedentary activities which included watching television, use of computer and video games, and duration of sleep/day of each study participant. In order to know regular habit of physical activity, we asked participants to report how long did they spend every day in a typical week doing activities which require physical activity. These activities included playing games such as football, cricket and other outdoor games, running in field and cycling. WHO recommends to spend at least 60 minutes of physical activity per day for children aged 5–17 years [23]. Since we conducted this study among school children who might be involved in physical activities during their school stay, we split this recommended duration of physical activity per day into two bouts, in school and at home. We assumed that splitting the physical activity habit into school and home would give us the opportunity to have better understanding of actual physical activity pattern of students and also to help determining if any particular habit serves as risk factor for overweight problem in children. We asked the respondents to report the actual physical activity habit both in school and at home and we added to find the duration of total physical activity. We recoded the response into following categories: <60 minutes and ≥60 minutes per day for total physical activity and no time, <30 minutes and ≥30 minutes per day for physical activity at home and school. Based on the median duration of sedentary activities per day of all participants, we categorized length of sedentary activities into two groups. We communicated over the phone with parents of each participant to obtain respondent’s (case or control child) birth weight, parental weight and height to estimate BMI, and highest level of education completed by mother.

### Data analysis

We derived descriptive statistics to determine the distribution of demographic information including age, sex, education level, sleeping time, mother’s highest level of education and BMI among case and control groups. For continuous data, we estimated mean (standard deviation, SD) if the data were normally distributed or estimated median (interquartile range, IQR) otherwise. In order to compare mean or median of variables between cases and controls, we used *t*-test or Wilcoxon-rank sum test, respectively. We used Z-test to compare proportions between cases and controls. Initially, we determined association between outcome (overweight/obese) and exposure variables using simple logistic model (unadjusted model). Any exposure variable in simple logistic model with a beta coefficient at significant level 0.3 was selected for the multiple logistic regression model (adjusted model) [24]. We considered a p-value of <0.05 as significant level in multiple logistic regression model.

### Ethical considerations

We obtained verbal informed consent to enroll students from the principals of respective schools followed by verbal consent from the parents of each participant before interviewing the participant. We obtained ethical clearance from BRAC University for conducting this study.

## Results

### Respondents’ profile

From October through November 2007, we enrolled 198 students in this study; 99 cases and 99 controls. The participants were selected from class 5 to class 10 in order to tally with specific age groups. The mean age of study respondents was 13.1 years (SD: 1.2). Of 198 respondents, 107 (54%) were male. The mean birth weight of respondents was 3.1 kilogram (SD: 0.8). The average sleeping time was 7.5 hours (SD: 1.0) and was similar across two groups. The median sedentary activities per day of all participants was 4 hours (IQR: 4–6). The median monthly household expenditure of respondents’ families was US$522 (IQR: 373–709). Eighty-five (44%) of 198 respondents had at least one overweight parent. One hundred twenty-four (62%) mothers reported to complete graduation degree or more (Table 1).

**Table 1:** Demographic characteristics of study participants at seven selected schools in Dhaka, Bangladesh 2007

**Characteristics**	**Control**	**Case**	**p value**
	**N = 99**	**N = 99**	
Age in years, mean (SD)	13.2 (0.1)	13.1 (0.1)	>0.05*
Male sex, n (%)	52 (53)	55 (56)	>0.05***
Participant’s class, n (%)			
Five	8 (8)	8 (8)	>0.05***
Six	16 (16)	16 (16)	>0.05***
Seven	27 (27)	24 (24)	>0.05***
Eight	27 (27)	29 (29)	>0.05***
Nine	20 (20)	21 (21)	>0.05***
Ten	1 (1)	1 (1)	>0.05***
Birth weight in kg, mean (SD)	3.2 (0.7)	3.0 (0.8)	>0.05*
Sleeping time/day, mean ((SD)	7.7 (1.0)	7.4 (1.0)	>0.05*
BMI, mean (SD)	18.5 (2.4)	28.8 (3.6)	<0.01*
Monthly family expenditure in US$, median (IQR)	522 (373–597)	522 (448–746)	>0.1**
At least one overweight parent^§^, n (%)	31 (32)	54 (56)	<0.00***
Maternal education			
Up to higher secondary, n (%)	35 (35)	34 (34)	>0.05***
Graduation degree and more, n (%)	60 (61)	64 (65)	>0.05***

*t-test; ** Wilcoxon-rank sum test; *** Z-test.

§ BMI of parents were assessed using self reported height (m) and weight (kg) of parents. BMI > 24.9 was considered as overweight.

### Risk factors associated with overweight and obesity

Unadjusted regression model revealed that three factors were independently associated with overweight or obesity in children (Table 2). Having at least one overweight parent and spending >4 hours each day on sedentary activities such as watching television and/or computer games were risk factors for overweight or obesity. Physical activity at home for ≥30 minutes each day was protective against childhood overweight or obesity. We did not find association between childhood overweight or obesity and duration of total physical activity for ≥60 minutes per day, duration of physical activity at school, sex, or maternal education level and these variables were not included in multiple logistic model. The multiple logistic regression analysis showed that children who had at least one overweight parent were nearly three times more likely to be overweight or obese (OR = 2.8; 95% CI: 1.5–5.2) compared to children whose parents were not overweight, and children who spent >4 hours on sedentary activities each day were two times more likely to be overweight or obese than children who spent less time on sedentary activities (OR = 2.0, 95% CI: 1.1–3.7). Children who spent ≥ 30 minutes each day with outdoor games at home that involved physical exercise had decreased odds (OR = 0.38, 95% CI: 0.1–0.8) of being overweight or obese compared to children who did not exercise at home (Table 2).

**Table 2:** Risk factors associated with overweight and obesity among school children in Dhaka, Bangladesh 2007

**Risk factor**	**Control**	**Case**	**Odds ratio (95% CI)**	
	**n (%)**	**n (%)**	**Unadjusted**	**Adjusted**
Having at least one overweight parent				
No	66 (68)	43 (44)	reference	reference
Yes	31 (32)	54 (56)	2.7 (1.5–4.8)*	2.8 (1.5–5.2)*
Total physical activities per day				
<60 minutes^†^	64 (65)	69 (70)	reference	Not included
≥60 minutes	35 (35)	30 (30)	0.80 (0.4–1.4) ^¶^	
Physical activities at home per day				
No	13 (13)	27 (27)	reference	reference
<30 minutes	28 (28)	27 (27)	0.5 (0.2–1.1)^§^	0.4 (0.2–1.0)^§^
≥30 minutes	58 (59)	45 (45)	0.4 (0.2–0.8)*	0.4 (0.2–0.9)*
Physical activities at school per day				
No	40 (40)	43 (43)	reference	
<30 minutes	26 (26)	22 (22)	0.8 (0.4–1.6) ^¶^	Not included
≥30 minutes	33 (33)	34 (34)	0.9 (0.5–1.8) ^¶^	
Sedentary activities				
0–4 hours	59 (60)	41 (40)	reference	reference
> 4 hours	40 (40)	58 (59)	2.1 (1.2–3.7)*	2.0 (1.1–3.7)*
Sex				
Female	47 (47)	44 (44)	reference	Not included
Male	52 (52)	55 (56)	1.1 (0.6–2.0) ^¶^	
Maternal education				
Up to higher secondary	35 (37)	34 (35)	reference	Not included
Graduation or more	60 (61)	64 (65)	1.1 (0.6–2.0)^¶^	
Sleeping time	99 (100)	99 (100)	0.8 (0.6–101)^¶^	Not included
Monthly household expenditure	99 (100)	99 (100)	1.0 (1.0–1.0) ^¶^	Not included

*p < 0.05; ^§^ p > 0.05; ^¶^p > 0.3.

^†^ This group included 5 controls and 13 cases who reported no physical activity.

## Discussion

Our data suggest that having overweight parent and engaging in sedentary activities including watching television and playing computer games for > 4 hours a day were potential risk factors for childhood overweight or obesity, whereas regular physical activity at home for at least 30 minutes seemed to be a protective factor. To our knowledge, risk factors for overweight and obesity among young children in low-income countries including Bangladesh have not been explored previously. The findings of this study are consistent with previous studies from high and middle-income countries [4-6,9].

Our data demonstrated that having at least one overweight parent increased the likelihood of a child being overweight, a finding consistent with previous studies in middle- and high- income countries such as Brazil and Australia [4,25]. Genetic factors are considered as one of the risk factors for overweight and obesity in children [25]. However, the prevalence of childhood overweight and obesity in the urban areas in Bangladesh has increased only in the last few years, which might be too short a time for any significant genetic changes in the population [2]. While we did not collect data about food consumption practice of the study respondents, fast food consumption habit has been previously found as a potential risk factor for overweight and/or obesity among children [26]. We suspect that it could be the family dietary habit that contributes to weight gain among family members including young children.

Our data suggest that children who spent greater time in physical activities such as playing general outdoor games were less at risk for being overweight or obese although the association was not significant. We also identified that physical activity in school was not associated with being overweight or obese. This lack of association indicates students’ tendency to be engaged in outdoor games in school. Since each of the study school had playing ground, the majority of students probably play sports during the games period. However, children who were less likely to be engaged in games at home or had little engagement time (<30 minutes) were nearly three times more likely to be overweight, a finding consistent with previous studies [5,9]. The reduced physical activity of children during home-stay could be linked with the rapid urbanization of Dhaka where around 15 million people are currently residing. The city is expanding in all directions in response to the need for housing, leading to a reduction of open recreation spaces and therefore, probably contributing to a change in physical activity pattern of both children and adults. However, reduced space issue remained doubtful as the healthy children of this study were engaged in physical activities while residing in the same location. Further exploratory research among young children to understand physical activity practices including type of activities practiced would be useful to design effective public health programs that aim to promote physical activity in this setting.

Technological advances in the form of hand-held electronic devices and computer games, and television programs have probably contributed to adopt a lifestyle that involves to less physical activity and more sedentary activity [7]. A study conducted among children in Iran reported an association between watching television and being overweight [5]. The study demonstrated that watching television decreased the amount of time spent on playing outdoor games which might resulted in gaining extra weight. Another study in the US reported that watching television or videos for >2 hours a day increased the risk of being overweight in children [27]. In this study, we combined the time spent on watching television and using computers per day, and found that overweight children spent more time (>4 hours) on sedentary activities compared to healthy children.

Studies conducted in Brazil and Iran have reported lower educational status of mother as a risk factor for overweight and obesity in children [4,5]. However, in this study we found that mothers of overweight and obese children were more educated than mothers of healthy children, although the association was not significant. One hypothesis to explain this contrasting finding could be related to respondents’ socioeconomic status. As the respondents were selected from the schools in middle class residential areas, more mothers were likely fall into the highly educated category (graduate and more).

### Limitations

Several limitations of this study should be noted. One important limitation was the relatively short duration, 2 months, for data collection which resulted in lower than estimated respondent enrollment which could reduce study power. However, the majority of our findings were consistent with previously published reports, suggesting that lower sample size likely had little impact on our assessment. We did not use any previously validated physical activity questionnaire to measure physical activity of study participants. Use of valid questionnaire could provide robust assessment of physical activity habit and it’s relationship with risk of overweight or obesity. However, the list of activities that we used to measure physical activity of children included those that Bangladeshi children used to do in this context. Yet, the importance of using validated physical activity questionnaire cannot be ignored. We did not collect data on individual games they played at home or in school rather we asked them to report us their engagement in the activities that we included in the list (e.g. football, cricket, and other outdoor games, running and cycling). Therefore, we could not inform if the activities done at home by the healthy children (controls) were likely to be of a higher intensity compare to overweight or obese children (cases). The cases were selected by teacher’s visual assessment. Therefore, it could happen that the most overweight children in the class were selected. However, as we found that the BMI of cases were normally distributed (data not shown), we suspect visual selection of cases might had modest impact on study outcome. Although it would be ideal to assess height and weight of parents, since the interviews were time-consuming and conducted after school hours, we communicated with each parent over the telephone and relied on self-reported anthropometric measurement of parents. Therefore, the possibility of error during measurement of height and weight remained valid. We conducted this study in seven schools in Dhaka city. So, the risk factors that we identified may not be representative of every urban city in Bangladesh.

## Conclusions

Our study demonstrated that several risk factors such as having overweight parent, limited physical exercise at home and high levels of sedentary activities are associated with overweight and obesity among urban school children in Dhaka, Bangladesh. Public health programs are warranted to increase awareness on these risk factors among children and adolescents in order to reduce the future burden of obesity-associated chronic diseases. Among different settings, schools should be the priority setting to target both children and adolescents because it offers momentous opportunities for prevention.

## Endnotes

^a^“Overweight children” included those who were obese.

## Competing interests

The authors declare that they have no competing interests.

## Authors’ contribution

MUB, SZ and TA designed the study. MUB coordinated data collection, extracted the data, performed statistical analysis, and drafted the manuscript. SZ and TA revised the manuscript and provided comments and suggestions. All authors read and approved the final version.

## Pre-publication history

The pre-publication history for this paper can be accessed here:

http://www.biomedcentral.com/1471-2431/13/72/prepub
